# Proteogenomic analysis prioritises functional single nucleotide variants in cancer samples

**DOI:** 10.18632/oncotarget.21339

**Published:** 2017-09-27

**Authors:** Shiyong Ma, Ranjeeta Menon, Rebecca C. Poulos, Jason W.H. Wong

**Affiliations:** ^1^ Prince of Wales Clinical School, Faculty of Medicine, UNSW Sydney, Sydney, Australia; ^2^ Present address: Centre for Research Excellence in Tuberculosis and the Marie Bashir Institute for Infectious Diseases and Biosecurity, The University of Sydney and Centre for Infectious Diseases and Microbiology – Public Health, Westmead Hospital, Sydney, NSW, Australia

**Keywords:** Proteogenomics, phosphoproteomics, RNA-seq, Jurkat, splicing factor mutation

## Abstract

Massively parallel DNA sequencing enables the detection of thousands of germline and somatic single nucleotide variants (SNVs) in cancer samples. The functional analysis of these mutations is often carried out through *in silico* predictions, with further downstream experimental validation rarely performed. Here, we examine the potential of using mass spectrometry-based proteomics data to further annotate the function of SNVs in cancer samples. RNA-seq and whole genome sequencing (WGS) data from Jurkat cells were used to construct a custom database of single amino acid variant (SAAV) containing peptides and identified over 1,000 such peptides in two Jurkat proteomics datasets. The analysis enabled the detection of a truncated form of splicing regulator YTHDC1 at the protein level. To extend the functional annotation further, a Jurkat phosphoproteomics dataset was analysed, identifying 463 SAAV containing phosphopeptides. Of these phosphopeptides, 24 SAAVs were found to directly impact the phosphorylation event through the creation of either a phosphorylation site or a kinase recognition motif. We identified a novel phosphorylation site created by a SAAV in splicing factor SF3B1, a protein that is frequently mutated in leukaemia. To our knowledge, this is the first study to use phosphoproteomics data to directly identify novel phosphorylation events arising from the creation of phosphorylation sites by SAAVs. Our study reveals multiple functional mutations impacting the splicing pathway in Jurkat cells and demonstrates potential benefits of an integrative proteogenomics analysis for high-throughput functional annotation of SNVs in cancer.

## INTRODUCTION

DNA sequencing technologies have enabled the rapid identification of single nucleotide variants (SNVs) within cancer genomes. Hundreds to thousands of non-synonymous SNVs can now be routinely identified within gene coding regions of individual cancers through whole genome or exome sequencing (WGS/WXS) [1], and expressed SNVs can also be detected by RNA sequencing (RNA-seq) [2]. To determine whether these SNVs are functionally important in cancer, *in silico* functional annotation tools are often used. This serves to reduce the total numbers of SNVs that are considered in downstream data analysis. However, experimental validation of the role that these SNVs have on protein structure and function remains a major challenge and therefore is not typically performed in routine analyses.

The detection of a SNV translated at the protein level is often the first step taken to infer whether the SNV is functional. Mass spectrometry-based proteomics experiments are now capable of detecting thousands of proteins in a single experiment. Recent studies have shown that by integrating SNV calls from WXS or RNA-seq data, it is possible to generate a sample-specific reference protein database that can be used to query a sample-matched proteomics dataset. For example, RNA-seq data from two colon cancer cell lines have been used to generate customised databases containing single amino acid variants (SAAVs) which can be applied to search the respective proteomics data [3]. This study identified an additional 37 and 17 peptides in the SW480 and RKO cell lines, respectively. More recently, Sheynkman et al. [4], similarly generated a customised database with SAAVs for the Jurkat cell line using RNA-seq data. This customised database enabled the detection of 395 SAAVs against a trypsin digested LC-MS/MS dataset. In a larger scale analysis by The Cancer Genome Atlas (TCGA) [5], 86 colorectal cancer proteomics datasets were searched against customised databases consisting of variants detected from sample-matched RNA-seq data. In this study, despite the large sample size, only 796 SAAVs were detected across 86 samples. A possible reason for this is that the RNA-seq data for TCGA colorectal cancer samples were not sufficiently deep for calling single nucleotide variants (SNVs) (~10M 50bp single end reads versus ~100M 100 bp paired-end reads for the Jurkat dataset from Sheynkman et al. [4]). Furthermore, the proteomics data was generally of lower depth than the Jurkat dataset (~100K spectra per sample versus ~500K spectra for the Jurkat dataset).

These studies have clearly demonstrated the ability to detect and quantify SAAV containing peptides. Nevertheless, how the detection of SAAVs at the protein level can benefit downstream analyses of protein function remains to be fully demonstrated. In this study, we first assess the potential of using an ‘ultra-deep’ Jurkat proteomics dataset to increase the number of SAAV containing peptides identified. We use several strategies to evaluate the accuracy of these SAAV containing peptides. Finally, to further demonstrate the potential of proteogenomics, we use a Jurkat phosphoproteomics dataset to show that this data can be used to directly identify SNVs that create novel protein phosphorylation events. Through this analysis, we highlight and discuss candidate SNVs that may play a role in the leukaemogensis of Jurkat cells.

## RESULTS AND DISCUSSION

### SAAV containing peptides detected in Mertins and Sheynkman jurkat datasets

**Figure 1:**
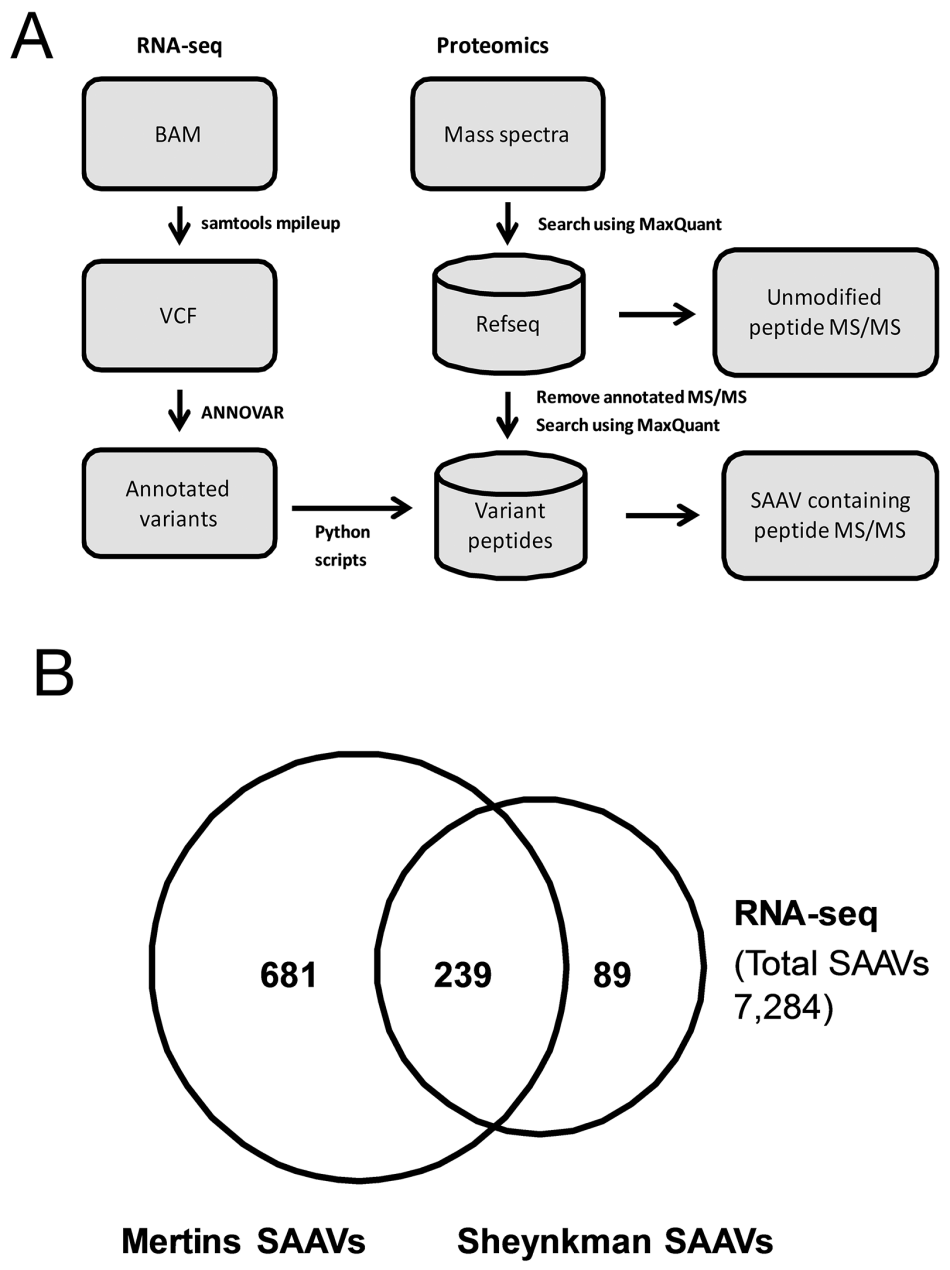
Analysis of RNA-seq variants in proteomics datasets **(A)** Schematic illustration of the RNA-seq/Mass spectrometry-based variant peptide detection pipeline. **(B)** Overlap of unique SAAVs detected in the Mertins and Sheynkman datasets.

A flowchart of the data analysis pipeline used is shown in Figure 1A. Of the 42,471 SNVs detected from the RNA-seq dataset, 7,284 were annotated as non-synonymous and 211 as stop gain substitutions (Supplementary Table 1). Using these SNVs, a customised protein database was generated and, along with the human reference proteome, was used to search the Sheynkman dataset (Supporting Data). In total, 378 SAAV containing peptides from 358 unique SAAVs were detected from the Sheynkman dataset (Supplementary Table 2). This is slightly less than the number of previously reported SAAVs containing peptides (n = 421) and unique SAAVs (n = 395) using the same data [4].

As the aim of this study was to identify functional SNVs, we examined how the use of higher depth proteomics data can increase the number of candidates. To achieve this, the Mertins dataset, which has over 2 million (i.e. 500%) more tandem mass spectra than the Sheynkman dataset, was analysed similarly. In total, 1,048 SAAV containing peptides, consisting of 929 unique SAAVs, were identified (Supplementary Table 3). The majority (72.9%, n = 239/328) of unique SAAVs detected from the Sheynkman dataset overlapped with the SAAVs detected from the Mertins dataset (Figure 1B). However, an additional 681 SAAVs could now be identified from the Mertins dataset. This represents an increase of almost three times as many SAAVs detected, when compared with the Sheynkman dataset (920 versus 328). Across both datasets, SAAV containing peptides consisted of 0.61% (378/62,204) and 0.70% (1,048/150,159) of the total peptides detected from the Sheynkman and Mertins datasets, respectively.

### Comparison between SAAV detection using variants from RNA-seq and whole genome sequencing (WGS) data

Variant calling using RNA-seq data can result in not detecting all variants in exonic regions since it is only able to detect SNVs in expressed genomic regions. Furthermore, studies have shown that RNA-seq data can also have higher false positive detection rates when compared with DNA sequencing due to mismatches arising from RNA to cDNA conversion during RNA sequencing library preparation as well as the complexities associated with precise alignment of reads spanning spliced junctions. To determine whether RNA-seq SNV detection is adequate, with regard to the goal of identifying SAAV containing peptides, we sought to compare the use of SNVs from RNA-seq and WGS.

**Figure 2:**
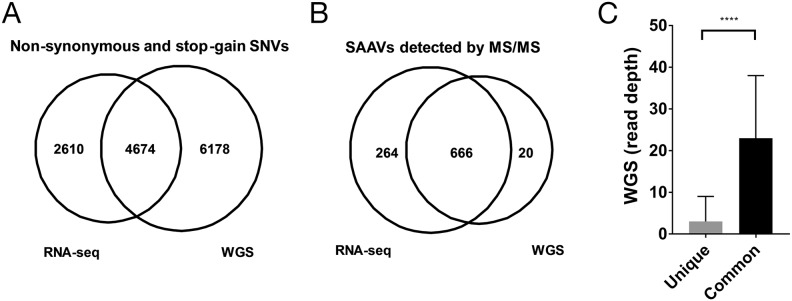
Comparison of RNA-seq and WGS variants for proteomics **(A)** Overlap of unique SAAVs detected in the Mertins dataset using RNA-seq SNVs and WGS SNVs. **(B)** Distribution of WGS read coverage of SAAVs that were unique to the RNA-seq SNV dataset or common to both the WGS and RNA-seq SNV datasets.

The genome of the Jurkat dataset was recently whole genome-sequenced at 60X coverage [6]. To enable a more direct comparison of the RNA-seq and WGS data, we only considered exonic regions (Methods). Within exonic regions, 79,601 SNVs were detected of which 10,852 were non-synonymous or stop-gain variants from the WGS data (Supplementary Table 4). When compared with the non-synonymous or stop-gain SNVs from the RNA-seq dataset, 4,674 SNVs overlapped, representing 64.1% of all RNA-seq SNVs (Figure 2A). The higher number of SNVs found across exonic regions in the WGS data compared with the RNA-seq data is expected as the RNA-seq data only covers transcribed regions.

To determine whether the higher number of variants from the WGS data would result in a greater number of SAAV peptides being discovered, we searched the Mertins proteomics dataset using a custom database generated using the WGS SNVs. Despite the larger search database, only 686 SAAVs could be identified using the WGS custom SAAV peptide database compared with 930 identified from the corresponding RNA-seq custom SAAV peptide database (Figure 2B). The overlap of SAAV peptides was substantial with 97.1% (666/686) of the peptides identified from the WGS custom SAAV peptide database also present in the search using the RNA-seq custom SAAV database. Examination of the genomic positions of the SNVs corresponding to the SAAV peptides identified that are unique to the custom RNA-seq database showed that the median coverage in the WGS data was just 3 reads and overall had significantly less coverage than SNVs corresponding to SAAV peptides common to both RNA-seq and WGS databases (Figure 2C, p < 0.0001 Mann-Whitney test). This suggests that, in this case, SNVs from RNA-seq data provides a better reference proteomics datasets compared with WGS, where read coverage is generally lower. In particular, RNA-seq read coverage is enriched within highly expressed transcripts, meaning that it would be more sensitive in detecting SNVs corresponding SAAV due to the higher protein abundance.

### Evaluation of SAAVs containing peptide identifications

To demonstrate that the SAAV containing peptides that we identified are true positives, we further performed a series of control analyses. Firstly, the SAAV containing peptides common to both RNA-seq and WGS SNV databases were classified as “heterozygous” or “homozygous” based on the variant allele frequency of the corresponding SNV derived from the WGS data. In total, we detected 274 “homozygous” (variant allele frequency (VAF) = 1.0) and 516 “heterozygous” SAAV containing peptides. Of the 274 “homozygous” SAAV containing peptides, we found 7 peptides with a corresponding reference peptide detected. This is unexpected as only a single sequence should be present at homozygous alleles. Further examination of the WGS sequence coverage showed that these SNVs had low coverage (alternate allele count < 10, Table 1). Examination of the corresponding RNA-seq data shows that these SNVs are in fact heterozygous with a variant allele frequency between 0.47 and 0.64 (Table 1).

**Table 1:** Homozygous SNVs where a reference peptide was identified by mass spectrometry

Alternate peptide	Reference peptide	Protein	Variant	RNA VAF (count)	WGS VAF (count)	Alternate intensity	Reference Intensity
**MVWDSQVSEHFFEYKK**	MVWDSQ**A**SEHFFEYKK	NM_001142677	p.A307V	0.634 (45)	1.00 (3)	122,530,000	29,178,000
**SQLPDLSAPHSYSPGR**	SQLPDLS**G**PHSYSPGR	NM_015114	p.G891A	0.615 (120)	1.00 (2)	469,400,000	339,370,000
**QVPPDLFQPYTEEICQNLR**	QVPPDLFQPY**I**EEICQNLR	NM_001619	p.I150T	0.535 (385)	1.00 (2)	22,980,000	96,743,000
**SGVHVVVTGPPNAGK**	SG**A**HVVVTGPPNAGK	NM_133644	p.V282A	0.506 (42)	1.00 (3)	797,570,000	64,056,000
**YHSLPPMYYR**	YHSL**A**PMYYR	NM_001252036	p.A86P	0.503 (94)	1.00 (5)	27,447,000	482,390,000
**TGSTLVQR**	TGS**A**LVQR	NM_002675	p.A331T	0.491 (26)	1.00 (9)	1,232,100,000	1,585,200,000
**ADGTPITQSVEMTTPSK**	ADGTPITQ**G**VEMTTPSK	NM_020070	p.G159S	0.471 (120)	1.00 (2)	220,560,000	140,030,000

In contrast, for the 516 “heterozygous” SAAV containing peptides, a much higher proportion of peptides (n=355) had the corresponding reference sequence detectable (Supplementary Table 5). The discrepancy between the number of reference sequence peptides corresponding to the SAAV containing peptides detected can be attributed to the stochastic nature of peptide sampling by mass spectrometers [7]. Nevertheless, the proportion of “heterozygous” SAAV containing peptides with the corresponding reference peptide detected was significantly higher (p < 0.0001, Fisher's exact test) than the “homozygous” SAAV containing peptides.

**Figure 3:**
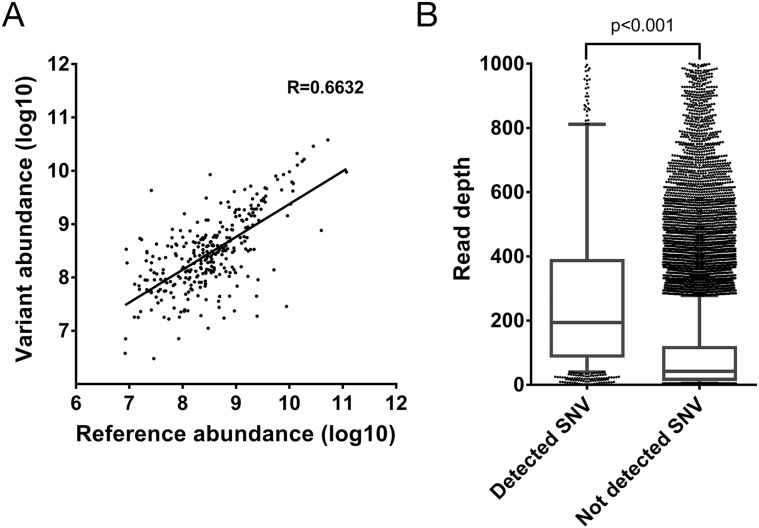
Evaluation of properties of SAAV containing peptides **(A)** Correlation between the abundance of SAAV containing peptides and its corresponding reference peptide from the Mertins dataset. A total of 467 peptides were paired in the comparison. **(B)** Comparison of the depth of RNA-seq read coverage of SNVs identified by the corresponding SAAV containing peptide by mass spectrometry and unidentified SNVs (p < 0.0001, Mann-Whitney test). Error bars represent 10-90 percentile.

The detection of the 355 reference peptides with a corresponding SAAV containing peptide allows the abundance of the corresponding peptides to be directly compared. Given that the corresponding SNVs are heterozygous, it would be expected that the abundance of the two alleles would be similar. Indeed, a clear correlation between the abundance of the reference and the corresponding SAAV containing peptide can be seen (R=0.6632, Figure 3A). This suggests that the two alleles of most heterozygous SNVs are co-transcribed and co-translated under the same regulatory mechanisms.

Finally, as the 1,010 SAAVs detected by mass spectrometry represent only 13.9% (n = 1,010/7,284) of all non-synonymous and nonsense SNVs detected by RNA-seq, we compared the read depth of SNVs corresponding to SAAV containing peptides with all remaining SNVs. The read depth in RNA-seq of identified SNVs was significantly higher than unidentified SNVs (median 194 versus 42 reads, p < 0.001, Mann-Whitney test) (Figure 3B). This suggests that SNVs that are in more highly expressed transcripts are more likely to be detected as SAAVs by mass spectrometry as the corresponding protein is also more abundant.

### Inclusion of variant peptides impacts protein quantification

Accurate protein quantification relies on the assumption that all proteolytic peptides are being detected and quantified. Standard database searches do not detect and include SAAV containing peptides in protein quantification. Thus, we examined whether the inclusion of SAAV containing peptides in the calculation of protein abundance may impact upon protein quantification accuracy. We used the Sheynkman dataset for this analysis, as the RNA-seq and proteomics data were generated from the same cells [8]. Using the Sheynkman dataset, we found good concordance between iBAQ and the RNA-Seq based expression values (R=0.68230).

**Figure 4:**
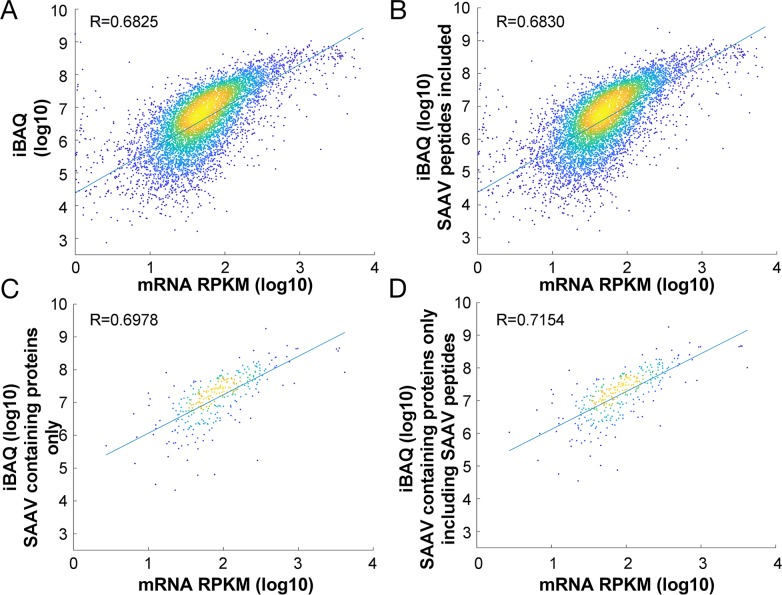
Impact of SAAV containing peptides on protein quantification Comparison of the correlation between protein abundance and mRNA expression in the Jurkat cell line before **(A)** and after **(B)** inclusion of SAAV containing peptides for all 6,881 proteins. Comparison of only 318 proteins that contain SAAVs before **(C)** and after **(D)** inclusion of the SAAV containing peptides.

In total, 378 proteins contained SAAV containing peptides. Correlation of the updated iBAQ values incorporating the intensity of SAAV containing peptides (see Methods) across all proteins (n = 6,881) with the corresponding mRNA expression value showed a modest but significant (p<0.01, Williams’ (1959) *t*-test) increase in correlation coefficient (R = 0.6825 to R= 0.6830; Figure 4A, 4B). To more clearly illustrate the impact of the updated iBAQ values, we calculated the correlation of the updated iBAQ values specifically for the 318 proteins with SAAV containing peptides. In this case, the mRNA expression value showed a more significant (p<0.0001, Williams’ (1959) *t*-test) increase in correlation coefficient, from R = 0.6978 to R = 0.7154 (Figure 4C, 4D). Although mRNA expression level is not an accurate measure of protein expression [9], the improved correlation that we observed over a pool of hundreds of proteins suggests that the inclusion of SAAV containing peptides in protein quantification may be beneficial to the accuracy of quantitative proteomics experiments.

### Truncation of YTHDC1 is a potential candidate cancer driver mutation in Jurkat cells

**Figure 5:**
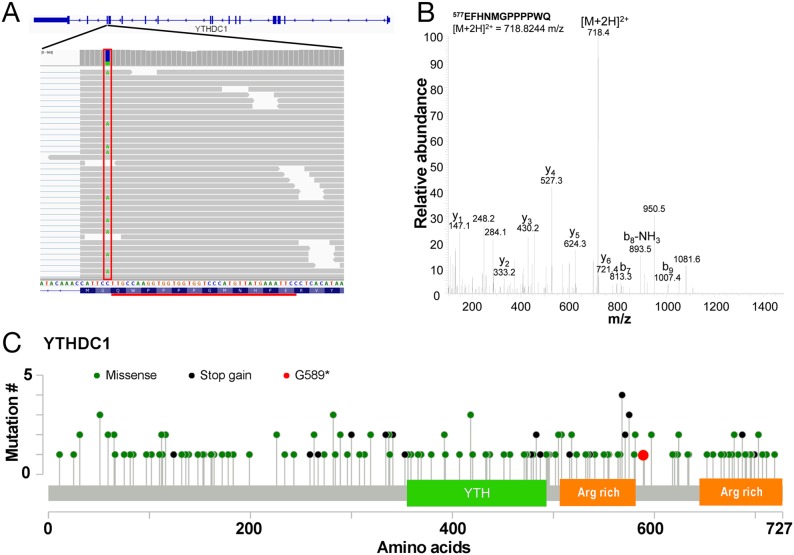
Truncation mutation in YTHDC1 **(A)** Illustration of the C>A/G>T SNV at chr4:69184242 (boxed in red) on the *YTHDC1* gene resulting in a G589^*^ SAAV. The tryptic peptide EFHNMGPPPPWQ detected as a result by mass spectrometry is underlined in black. **(B)** Representative tandem mass spectrum of the doubly charged ion of EFHNMGPPPPWQ. **(C)** Mutation map of COSMIC mutations across YTHDC1 showing the YTH domain and arginine (Arg) rich regions.

In order to determine whether the use of proteomics data can help the identification of candidate cancer driver mutations, we sought to prioritise SNVs that are most likely to alter protein function. Examination of the list of SAAVs (Supplementary Table 2 & 3) reveals that there is only one stop gain event that can be detected across both the Sheynkman and Mertins datasets. This SAAV results in the truncation of YTHDC1 at G589 (Figure 5A) and the identification is supported by manual examination of the tandem mass spectrum (Figure 5B). YTHDC1 is a regulator of alternative splicing that specifically recognises and binds N6-methyladenosine (m6A)-containing RNAs [10–12]. The truncation of YTHDC1 removes an arginine-rich region at the C-terminus of the protein (Figure 5C). As arginine-rich motifs in proteins are generally known to be important in RNA binding [13], this truncation may impact the RNA binding ability of YTHDC1.

Based on the RNA-seq data, the variant is heterozygous (Supplementary Table 5) but is completely absent from the Exome Aggregation Consortium (ExAC) database [14], suggesting that it is likely to be a somatic mutation in the Jurkat cell line. Examination of known somatic mutations in *YTHDC1* from the Catalog of Somatic Mutations in Cancer (COSMIC) database shows a relatively large number of stop gain mutations over the protein (Supplementary Table 6). Of all COSMIC samples with point mutations in *YTHDC1*, 14.1% (24/170) of mutations are stop-gain mutations which are significantly more than in all genes in general (p < 0.0001, chi-square test, Supplementary Table 6). Many of the truncations occur within <25 amino acids of the G589^*^ mutation (Figure 5C). Tumour suppressor genes tend to have a relatively high percentage of stop gain mutations [15]. For instance, the percentage of substitution mutations that are stop-gain for some well-known tumour suppressor genes such as *MLH1*, *PTEN* and *VHL* are 13.6% (37/272), 29.3% (657/2,242) and 20.3% (250/1,229), respectively. While, to our knowledge, *YTHDC1* mutations have not previously been implicated in leukaemia, mutations affecting splice factors are common in leukaemia [16, 17] and myelodysplastic syndromes [18]. Thus, the G589^*^ stop gain mutation in YTHDC1 may contribute to leukaemogenesis in Jurkat cells by impacting the RNA splicing pathway.

### Novel phosphorylation sites created by SNVs

Having established that potential cancer-associated SNVs can be inferred from proteomics data, we further investigated the use of phosphoproteomics data in more directly identifying SNVs that can alter the phosphorylation state of a protein. Using the SAAV peptide database, we applied MaxQuant [19] to analyse the Mertins phosphoproteomics dataset (Table 2). In total, we identified 462 MS/MS spectra of SAAV containing phosphopeptides, representing 1.11% of the total phosphopeptide MS/MS spectra detected in the dataset (462/41,698) (Supplementary Table 7).

**Table 2:** Summary of datasets used in study

Experiment	Details	Reference
RNA-seq	~ 100 M, 100 bp PE reads	[8]
Whole genome sequencing	~900 M, 150 bp PE reads	[6]
Proteomics deep(Sheynkman proteomics dataset)	~ 0.5 M spectra	[8]
Proteomics ultra-deep(Mertins proteomics dataset)	~ 2.5 M spectra	[25]
Proteomics ultra-deep PTM(Mertins phosphoproteomics dataset)	STY(p) - ~ 0.85 M spectra	[25]

STY(p) – phosphorylation of S, T or Y

The inclusion of SAAVs within the search space can influence the detection of different phosphorylation events. For example, (1) the SAAV may create an amino acid that allows a novel phosphorylation site to form, (2) the SAAV may create a motif that allows a novel phosphorylation event to take place, and (3) the SAAV may not impact the formation of the phosphorylation event, but instead may simply add the SAAV into the protein database, thus allowing the SAAV-containing phosphopeptide to be detected. To this end, the phosphopeptides were examined further to determine to which of the three categories described above, each one belonged. In this case, to determine motif creation, we only considered proline-directed kinases such as mitogen-activated protein kinases (MAPK) and cyclin-dependent protein kinases (CDK) [20] as these have a simple and clearly defined phosphorylation motif ([S/T]P). As such, if a SAAV creates a proline that is immediately C-terminal of a serine or threonine, we considered that the gain of this proline was responsible for the phosphorylation of the N-terminally adjacent serine or threonine.

**Figure 6:**
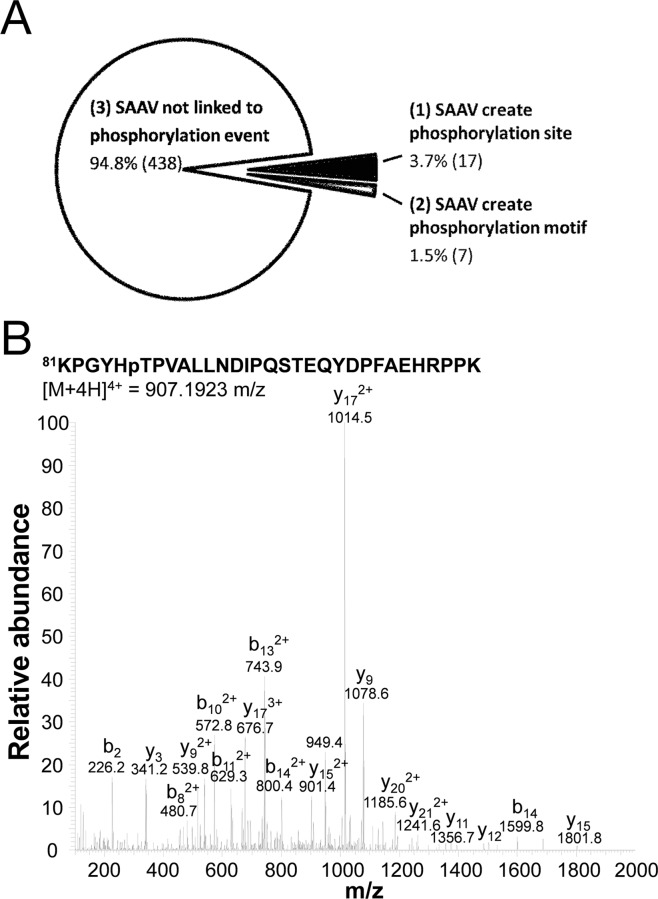
Phosphorylation sites created by SNVs **(A)** Phosphopeptides that were detected as a result of the inclusion of SAAVs into the search database, classified into one of 3 categories including, no impact of phosphorylation site, creation of phosphorylation site and the creation of a phosphorylation motif. **(B)** Tandem mass spectrum of quadruply charged SF3B1 tryptic phosphopeptide KPGYHpTPVALLNDIPQSTEQYDPFAEHRPPK containing the A85T SAAV.

Based on the 3 categories outlined above, each of the 462 SAAV phosphopeptides was categorised. We found that only a minority (5.2%, 24/462) of SAAVs created a phosphorylation event, whereas the majority simply enabled the detection of an existing phosphorylation event (Figure 6A). A summary of the SAAVs that created a phosphorylation site or a phosphorylation motif is shown in Table 3. Of the 24 SAAV sites that either created a phosphorylation site or a phosphorylation, only five do not correspond to known SNPs and are therefore likely to be somatic mutations (Table 3). One of these five putative somatic SAAVs results in the phosphorylation of T86 in the splicing factor 3b subunit 1 (SF3B1) protein. Manual examination of the tandem mass spectrum further confirmed the identification of this phosphopeptide (Figure 6B).

**Table 3:** List of phosphopeptides that have been detected as a result of a gain of phosphorylation site (grey), or a gain of a phosphorylation motif (white). Phosphorylation residue is indicated by bold and the SAAV residue is underlined

Gene	SAAV	Peptide	Genomic coordinates (hg19)	dbSNP ID	ExAc allele freq.
*ATP5A1*	p.A126S	VGLK**S**PGIIPR	chr18:43669656	NA	0.0000
*SGK223*	p.G1222S	QKPG**S**TPNLQQK	chr8:8176221	rs13269488	0.5286
*TANC1*	p.N250S	SGSSLEWNKDG**S**LR	chr2:160019863	rs12466551	0.9907
*TADA2A*	p.P6S	LG**S**FSNDPSDKPPCR	chr17:35771468	rs7211875	0.8597
*ATP5SL*	p.N40S	LGAAVAPEG**S**QKK	chr19:41944237	rs2231940	0.4053
*GBF1*	p.G1690S	GG**S**PSALWEITWER	chr10:104140350	rs11191274	0.0972
*PCM1*	p.N159S	DAST**S**PPNRETIGSAQCK	chr8:17796382	rs41364448	0.7324
*PCM1*	p.N159S	SKDAST**S**PPNRETIGSAQCK	chr8:17796382	rs41364448	0.7324
*SPDL1*	p.L586S	**S**HPILYVSSK	chr5:169031150	rs3777084	0.6613
*DDX27*	p.G766S	A**S**PSFEER	chr20:47859217	rs1130146	0.3498
*GPANK1*	p.A78S	IMK**S**PAAEAVAEGASGR	chr6:31632024	NA	0.0000
*SF3B1*	p.A86T	KPGYH**T**PVALLNDIPQSTEQYDPFAEHRPPK	chr2:198285797	NA	0.0000
*MEX3A*	p.A430T	SPT**T**SAGPELAGLPR	chr1:156046640	NA	0.0000
*LMO7*	p.M1162T	YLDQIGN**T**TSSQRR	chr13:76423248	rs7986131	0.7203
*RMI1*	p.N455S	**S**SQISNENDCNLQSCSLR	chr9:86617265	rs1982151	0.7152
*ICE1*	p.C391S	NSAE**S**VSEDDTTESQNYFGSLR	chr5:5460619	rs2619844	0.6964
*LAT*	p.P253S	TEPAALSSQEAEEVEEEGA**S**DYENLQELN	chr16:29001301	NA	0.0000
*PNPLA6*	p.A403P	ETPGRPPDPTGAPLPGPTGDPVKPTSLETP**S**PPLLSR	chr19:7606908	rs17854645	0.1651
*DMXL2*	p.S1288P	FGDTEAD**S**PNAEEAAMQDHSTFK	chr15:51791559	rs12102203	0.4925
*KIAA0586*	p.L703P	EA**S**PPPVQTWIK	chr14:58937416	rs1748986	0.9916
*GGA2*	p.A424P	NLLDLL**S**PQPAPCPLNYVSQK	chr16:23489711	rs1135045	0.7564
*ZNF235*	p.H296P	SPACS**T**PEKDTSYSSGIPVQQSVR	chr19:44792701	rs2125579	0.5206
*BRIP1*	p.S919P	YS**T**PPYLLEAASHLSPENFVEDEAK	chr17:59763347	rs4986764	0.5972
*SHF*	p.A242P	ETATQPLPLYDTPYEPEEDGA**T**PEGEGAPWPR	chr15:45467540	rs1632144	0.9763

SF3B1 is involved in the regulation of splicing as part of the splice factor complex. SF3B1 is a known phosphoprotein [21] and the phosphorylation of SF3B1 has also been shown to be associated with splicing activation [22]. However, little is known about how phosphorylation of sites in SF3B1 alters its activity. It may be possible that the phosphorylation of T86 that we identified modulates the activity of SF3B1. As with the stop-gain SNV in *YTHDC1*, analysis of the allele frequency of this *SF3B1* SNV in the ExAc database [14] shows that it is not present in the general population (Table 3) further suggesting this SNV is a somatic mutation.

## CONCLUSIONS

SNVs that lead to the creation of novel phosphorylation sites have previously been described in several studies. For example, the germline R405S variant in androgen receptor (AR) has been shown to result in the phosphorylation of S405 and this post-translational modification results in the disruption of protein-protein interactions between AR and EP300 [23]. In the context of cancer, *in silico* methods have been applied to predict the gain of phosphorylation from somatic mutations [24]. However, to our knowledge, ours is the first study to have used phosphoproteomics data to directly identify novel phosphorylation events arising from the creation of phosphorylation sites by SAAVs.

In summary, by integrating RNA-seq and proteomics data, we have shown that the translation of some SNVs can be detected at the protein level, and this can allow more accurate inference to the functional impact of mutations. Although our analysis does not enable direct inference of whether particular variants play a direct role in causing cancer, we have demonstrated that it can be used to further prioritise candidate SNVs. In this case, we found two SNVs which both affect the RNA splicing pathway in Jurkat cells. As splicing factor mutations are common in blood disorders [16–18], these mutations may play an important role in the leukaemogenesis. Uniquely, out study has also demonstrated that the integration of RNA-seq data with phosphoproteomics data can be used to directly discover novel phosphorylation events. With massively parallel sequencing and proteomics becoming increasingly commonplace, such analyses will likely become increasingly useful for cancer research.

## MATERIALS AND METHODS

### Data sources

The Jurkat (T-cell acute lymphocytic leukaemia) cell line RNA-seq data [8] were obtained from Gene Expression Omnibus (GSE: GSE45428) in SRA format. Matched deep LC-MS/MS Jurkat proteomics dataset [8] in RAW format were obtained from PeptideAtlas under the accession PASS00215. This is referred to in the manuscript as the Sheynkman proteomics dataset. Both ‘Ultra-deep’ and phosphorylation-enriched Jurkat proteomics datasets [25] were obtained in RAW format from MassIVE (MSV000078509). These are referred to in the manuscript as the Mertins proteomics dataset and phosphoproteomics dataset, respectively. Variant calls and aligned Jurkat whole genome sequencing data [6] were obtained from Zenodo (400615) and the NCBI Sequence Read Archive (SRA) (SRP101994), respectively. A list of datasets used in this study, together with their properties and sources are summarised in Table 2. COSMIC [26], ExAc [14] and dbSNP databases were used to assess SNV frequency and distribution.

### Variant calling from RNA-seq and WGS dataset

The Jurkat RNA-seq data in SRA format were converted to fastq, trimmed using sickle (version 1.33) and aligned using tophat (version 1.4.0) [27]. Samtools mpileup [28] was used for variant calling from the RNA-seq data. A variant filter was applied where SNVs with less than 2 reads and a mapping quality score of less than 10 were removed. Furthermore, to allow for a direct comparison of the variants called within exonic regions of the human genome, only variants that were present within exons, as defined by the RefSeq database, were kept for further analysis. This resulted in the detection of 42,471 SNVs. For the WGS data, only variant calls that were annotated as PASS and overlapped the RefSeq exons were used for further analysis. This resulted in 79,601 SNVs. To determine the reference and alternative allele count in the WGS dataset, samtools mpileup was used on the WGS BAM file obtained from SRA as described above.

### Generation of proteomics databases

To facilitate simple and accurate mapping of SNPs to SAAVs in proteins, the human Refseq protein annotations were used, as both the human proteome database, and for variant annotation. Annotation of SNPs against Refseq was performed using ANNOVAR [29]. To create the human Refseq proteome database in fasta format, the RefFlat GTF file was obtained from the Illumina iGenomes resource and the gffread utility (https://github.com/gpertea/gffread) was used to translate the transcripts defined in the GTF file into protein sequences.

In order to generate the custom SAAV database, a Python script was written to directly map SAAVs from the ANNOVAR annotated SNPs and to output theoretical tryptic peptides up to 1 missed cleavage either side of the SAAV.

### Processing of proteomics data

LC-MS/MS data in RAW format were analysed using MaxQuant (version 1.3.5.8) [19]. The data was searched using a “two-stage” strategy [30], where the LC-MS/MS data was first searched using human Refseq proteome and contaminant proteins and then subsequently, the remaining unidentified spectra were searched using the RNA-seq or WGS custom SAAV database. At each stage, a reversed decoy database was included for false discovery estimation. For all searches, carbamidomethyl (C) was selected as a fixed modification while oxidation (M) and acetylation (protein N-term) were selected as variable modifications. For searching of the phosphoproteomics dataset, phosphorylation (STY) was selected. For all searches, the mass tolerance was set at 10 ppm and false discovery rate set at 0.01. In the case of the phosphoproteomics dataset, only phosphorylation sites with a localisation score of ≥ 0.75 were retained for further analysis.

### Protein quantitative analysis

For protein quantitative analysis, the peptide ion intensity and the iBAQ quantitative value were obtained from MaxQuant. To calculate new updated protein iBAQ values incorporating SAAV containing peptides, the ion intensity of SAAV containing peptides was added to the respective protein's total ion intensity. The updated iBAQ value was calculated by dividing the updated total intensity value by the number of theoretically detectable peptides.

For the comparison of RNA expression to protein abundance, RNA expression was measured as Reads Per Kilobase of transcript per Million mapped reads (RPKM). To compare the significance of the change in the correlation of RNA and protein abundance before and after inclusion of SAAV containing peptides, significance was determined using the Williams’ (1959) *t-*test, calculated with the R package cocor [31].

## SUPPLEMENTARY MATERIALS FIGURES AND TABLES





## Abbreviations

COSMIC: Catalog of Somatic Mutations in Cancer
ExAC: Exome Aggregation Consortium
RNA-seq: RNA Sequencing
RPKM: Reads Per Kilobase of transcript per Million mapped reads
SNV: Single Nucleotide Variants
SAAV: Single Amino Acid Variants
TCGA: The Cancer Genome Atlas
WXS: Whole Exome Sequencing
WGS: Whole Genome Sequencing
