# Supplementary material for: Proteogenomic analysis prioritises functional single nucleotide variants in cancer samples
Source: Oncotarget. 2017 Sep 27;8(56):95841–52. doi: 10.18632/oncotarget.21339 (PMC5707065; doi:10.18632/oncotarget.21339)
Supplement: Supplementary file 1 [file oncotarget-08-95841-s001.pdf]

Proteogenomic analysis prioritises functional single nucleotide variants in cancer samples

SUPPLEMENTARY MATERIALS

Supplementary Tables containing lists of all annotated variants detected from the RNA-seq experiment (Supplementary Table 1), list of all SAAV containing peptides from the Sheynkman Jurkat proteomics dataset (Supplementary Table 2), list of all SAAV containing peptides from the Mertins Jurkat proteomics dataset (Supplementary Table 3), lists of all annotated variants detected from the WGS data within RefSeq exonic regions only (Supplementary Table 4), lists of variants detected from the RNA-seq and WGS data showing the corresponding reference peptide sequence where detected (Supplementary Table 5), list of all SAAV containing peptides from the Mertins Jurkat phosphoproteomics dataset (Supplementary Table 6), mutation frequencies of different substitution classes of specific genes and whole genome from COSMIC (Supplementary Table 7) (XLSX).

Supporting Data includes:

Fasta file of Jurkat SAAV containing peptides from RNA-seq and WGS used for database searching (TXT).

Supplementary Table 1: Lists of all annotated variants detected from the RNA-seq experiment.

See Supplementary File 1

Supplementary Table 2: List of all SAAV containing peptides from the Sheynkman Jurkat proteomics dataset.

See Supplementary File 1

Supplementary Table 3: List of all SAAV containing peptides from the Mertins Jurkat proteomics dataset.

See Supplementary File 1

Supplementary Table 4: Lists of all annotated variants detected from the WGS data within RefSeq exonic regions only.

See Supplementary File 1

Supplementary Table 5: Lists of variants detected from the RNA-seq and WGS data showing the corresponding reference peptide sequence where detected.

See Supplementary File 1

Supplementary Table 6: List of all SAAV containing peptides from the Mertins Jurkat phosphoproteomics dataset.

See Supplementary File 1

Supplementary Table 7: Mutation frequencies of different substitution classes of specific genes and whole genome from COSMIC.

See Supplementary File 1
